# A Review on Case Burden of Diabetes Mellitus Before and After the Implementation of National Programme for Prevention and Control of Cancer, Diabetes, Cardiovascular Diseases and Stroke

**DOI:** 10.7759/cureus.49446

**Published:** 2023-11-26

**Authors:** Yash Bhagwat, Sunil Kumar

**Affiliations:** 1 Medicine, Jawaharlal Nehru Medical College, Datta Meghe Institute of Higher Education and Research, Wardha, IND; 2 Internal Medicine, Jawaharlal Nehru Medical College, Datta Meghe Institute of Higher Education and Research, Wardha, IND

**Keywords:** diabetes screening, non-communicable diseases epidemiology public health community-based study, cross sectional study, diabetes mellitus, npcdcs

## Abstract

This article focuses on the role of the National Health Program called National Program for Prevention and Control of Cancer, Diabetes, Cardiovascular Disease, and Stroke (NPCDCS) in the screening and reduction of the case burden of Diabetes mellitus. The article first discusses the case burden of Diabetes before the implementation of NPCDCS and then the burden of the disease after the implementation of the program by mainly reviewing the cross-sectional studies done in four districts, Jaipur district, Gandhinagar district, Belagavi taluka district, and Udupi district. The studies were conducted at least four years after the program's implementation. The reason for preparing this review article is to assess the efficacy of NPCDCS in controlling the most dreaded chronic disease, which has its highest prevalence in India. Over the past century, there has been a consistent rise in the prevalence of Diabetes.

In all departments of medicine, Diabetes has been a common predisposing factor in several adversities such as blindness, limb amputation, cerebrovascular stroke, diabetic nephropathy, and other microvascular and macrovascular diseases. The studies include field-level cross-checking and on-ground cross-sectional studies, which were done in 2019 in Jaipur, standard cross-sectional studies from the primary data collected from the primary health care center in the Belagavi taluka district, a national-level cross-sectional study conducted by the National NCD Monitoring survey, and cross-sectional studies in Udupi district in Karnataka, which was the first district to be included by NPCDCS in its second phase.

## Introduction and background

Diabetes stands as a significant contributor to conditions like blindness, stroke, heart attacks, and lower limb amputation, according to the World Health Organization (WHO). Notably, there has been a 3% rise in diabetes-related mortality between 2000 and 2019 [1]. Diabetes mellitus is a chronic ailment characterized by elevated blood glucose levels. It emerges through two mechanisms: inadequate insulin production (Type 1) and diminished cell responsiveness to insulin (Type 2) [2].

In Type 1 diabetes, islet-specific antibodies form, involving CD4+ cells of the T cell-mediated immune response. Consequently, insulin production decreases, and hyperactive alpha cells increase glucagon secretion in Type 1 Diabetes Mellitus (T1DM) patients [3]. Conversely, Type 2 Diabetes Mellitus (T2DM) indicates beta cell dysfunction resulting from intricate interactions of environmental factors and biochemical pathways [4]. Obesity, hypertension, and hyperlipidemia emerge as key risk factors for T2DM. Excessive Free Fatty Acids (FFA) and hyperglycemia trigger beta cell dysfunction via ER (Endoplasmic Reticulum) stress, inducing apoptotic unfolded protein response [5]. Aberrations in insulin synthesis, its precursors, or secretion mechanisms lead to secretory dysfunction, culminating in beta cell failure and fostering T2DM development [6]. Disturbing epidemiological figures project a worrisome T2DM future. The International Diabetes Federation (IDF) reported nearly 4.2 million diabetes-related deaths in 2019, with over 463 million adults aged 20 to 80 living with diabetes [7]. Both hereditary and environmental factors contribute to T2DM. While genetic factors account for only 10% of genetic variance, environmental factors like sedentary lifestyle, high-calorie intake, and less physical activity play a more dominant role [8].

Diabetes also escalates the risk of coronary heart disease (Hazard Ratio {HR} - 2.00), ischemic stroke (HR- 2.27), and other vascular issues (HR-1.73) [9]. Risk factors for Diabetes encompass non-modifiable (genetics, age, etc.) and Modifiable (obesity, sedentary lifestyle, etc.) factors. The non-modifiable risk factors include genetics, family history, racial or ethnic traits, history of gestational diabetes, and increasing age (more than 45) [10]. Obesity, particularly with a Body Mass Index (BMI) exceeding 30, is a potent risk factor, though the obesity-T2DM relationship remains complex [11]. Sedentary behavior also heightens diabetes risk, with a 56% decrease in T2DM risk noted in participants walking 2-3 hours weekly [12]. WHO's 2015 Country Capacity Survey revealed that over 70% of countries have national health programs for diabetes prevention and management, but less than half are fully operational. Crucial glucose-lowering drugs are available only in half of primary healthcare facilities [13].

In India, the National Program for Prevention and Control of Cancer, Diabetes, Cardiovascular Disease, and Stroke (NPCDCS), implemented in 2010, spearheads diabetes prevention and management. In India, the NHP for diabetes prevention and management is the NPCDCS, which was implemented in 2010 [14]. It emphasizes early non-communicable disease detection, health awareness, healthy lifestyle promotion, affordable diagnosis, treatment, and rehabilitation [15]. Diabetes diagnosis relies on tests like Fasting Plasma Glucose (>126 mg/dl), HbA1c levels (≥6.5%), two-hour Oral Glucose Tolerance Tests, and random glucose plasma levels (≥200 mg/dl). Further tests for Diabetes etiology are not routine [16].

## Review

Methodology

**Figure 1:**
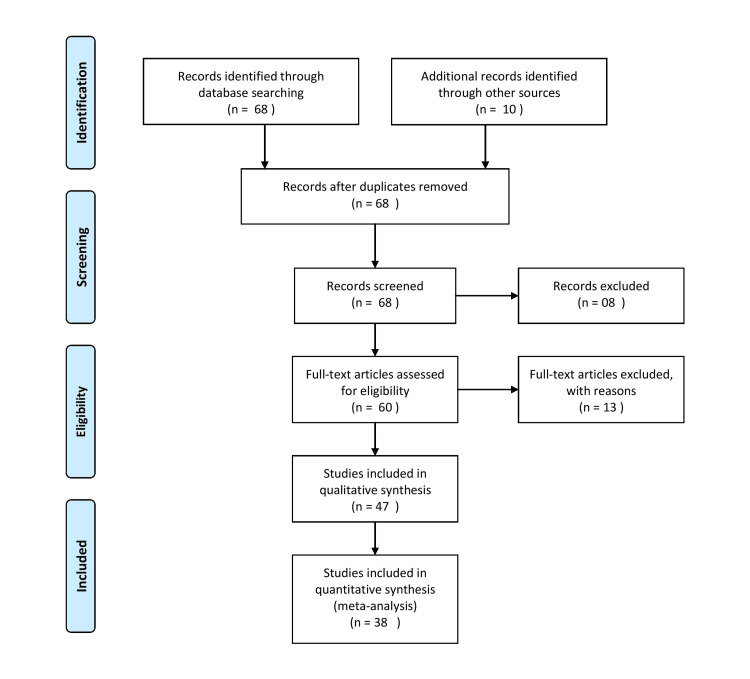
The selection process of articles used in the study Preferred Reporting Items for the systemic Review and Meta-Analysis (PRISMA) flowchart for the keywords which have been used in review of literature.

The search for literature in English was carried out using the electronic databases PubMed, ResearchGate, Google Scholar, and MEDLINE. The search terms were "Diabetes" or "NPCDCS" or "screening" or "NCD Monitoring Survey" or "Belagavi Taluka Cross Sectional Study" OR "Sedentary lifestyle" OR "lifestyle modifications". The authors' expertise and background in the topic helped assist with archiving relevant publications. The articles that match the following criteria are included in this review article: articles in English are included, articles on Diabetes pathophysiology are included as well, articles on NPCDCS, Studies on screening of Diabetes and other non-communicable diseases, studies of National NCD Monitoring Survey and WHO (World Health Organization) Reports on case burden of Diabetes are included. The articles excluded are the articles that lack full text of the publication, the language of the publication different than English, the sort of publication that differs from that of the illness in review, malignancy, co-morbidities with the diseases impacting presentation, specific treatment plan, or systematic reviews, meta-analyses, or empirical studies published in peer-reviewed scientific journals. The Preferred Reporting Items for Systematic Reviews and Meta-Analyses (PRISMA) method has been applied in the research methodology, as depicted in Figure 1.

Rapidly increasing case burden of diabetes in the decade 2000-09

The prevalence of Diabetes has been steadily increasing over the years in the last century. The prevalence of the disease has been rapidly increasing since the year 2000 [17]. In the early 1970s, the prevalence of the disease in urban areas was 2.3% and 1.5% in rural areas [18]. From 1971 to 2000, the reported cases increased ten-fold [19]. In the year 2002-03, the incidence of Diabetes was 8.4% among industrial workers and their families; new cases were detected with the fasting plasma glucose level test during screenings. The increase in the case incidence of the disease was steady in the mid-2000s [20].

Major risk factors were obesity, high adiposity in the upper body, physical inactivity, and psychological stress. Urban environment increases the likelihood of obesity and unhealthy diet. Studies have shown that the Indian population possesses some peculiar features that make them susceptible to Diabetes, such as low-risk thresholds. These risks include a normal range of adiposity in the body, which is >23 kg/m2. Presence of high central obesity even in the presence of normal or high insulin resistance [21]. In the post-urbanization period, there were several changes in lifestyles. The introduction of machinery also reduced the amount of physical labor and led to a more sedentary work environment. NPCDCS focuses on prevention, early detection, and management, emphasizing lifestyle changes, awareness drives, and healthcare professional training. The initiative provided diagnostic and treatment aid by integrating NCD (Non-Communicable Diseases) services into the existing healthcare framework. NPCDCS targeted the reduction of NCD prevalence, enhanced patient outcomes, and reduced strain on healthcare resources. It employed community involvement and policy interventions to foster healthier lifestyles, ultimately curbing NCD-linked morbidity and mortality [22]. The case burden in 2000 was counted at 32 million. Following this, the count at the end of 2007 was 41 million, following this, at the end of 2010, the number reached 51 million [23].

The initiatives of NPCDCS and the change in case burden after it's implementation

The NPCDCS was started by the Government of India in 2010 as part of the 11th five-year plan. This initiative drew inspiration from the 7th five-year plan introduced in 1987, piloted in select districts of Jammu Kashmir, Karnataka, and Tamil Nadu [24]. The core objectives of the NPCDCS encompassed various aspects: identifying individuals at high risk of Diabetes and intervening early through effective health education, prompt diagnosis, and timely treatment. These measures aimed to curtail mortality and morbidity rates, particularly among the vulnerable demographic groups. Furthermore, the program aimed to prevent complications associated with diabetes, encompassing metabolic conditions cardiovascular, renal, and ocular issues. Another key focus was to ensure equal opportunities for physical accomplishments and academic success for individuals living with Diabetes. Additionally, the NPCDCS sought to provide rehabilitation services for those who were disabled or handicapped [25].

To address the underlying determinants of the disease such as hypertension, obesity, stress, elevated blood lipid levels, and alcohol consumption, a crucial step was generating public awareness. To achieve this, a range of awareness campaigns were rolled out at the community level with the support of community-based organizations, panchayats, and through Interpersonal Communications (IPC). Screening and early detection of Diabetes were carried out through opportunistic and camp-based screening approaches. Collaborative efforts with other programs like the National Program for Health Care of Elderly (NPHCE), the National Tobacco Control Program (NTCP), and the National Mental Health Program (NMHP) were instrumental in implementing effective treatment and rehabilitation practices [26].

A comprehensive assessment of the NPCDCS's performance for 2019-20 was carried out in a specific study conducted in Jaipur, Rajasthan. This descriptive observational study was conducted at a community health center and involved on-the-ground screening activities in evaluating the program's impact [27]. This field-level cross-checking aimed to identify any disparities or gaps between the reported and actual screening activities. A random sample of 200 families was selected, and their recent screening history for Non-Communicable Diseases (NCDs) within the past year was verified. Moreover, the awareness levels of hospital staff and Community Health Center (CHC) workers regarding NCDs and NPCDCS were evaluated. Data collection relied on a questionnaire-based approach. Supplementary data was sourced from Form 1 and Form 3 of the NPCDCS, encompassing all subcenters, the Out Patient Department (OPD), and the NCD register. The cross-checking analysis disclosed that 38.27% of individuals screened tested positive for Diabetes. Surprisingly, a substantial portion (approximately 17.5%) of these cases were not officially reported under NPCDCS [28].

Another study was conducted in Belagavi taluka in 2018. The primary data was collected from the primary health center (PHC). This was a cross-sectional study; 36 subcenters were selected with a population of 248,753 under the study. The implementation of NPCDCS was found to be 100% in all subcenters and health check-up camps for the over 30 screening for NCDs. Out of the 64,096 individuals, 1608 were screened positive for Diabetes. It was found that the prevalence rate of Diabetes was only 2.62 %. Out of the 3124 positive patients for Diabetes, 1412 (45.25%) were newly found cases [29]. A national-level cross-sectional study was conducted as a National NCD Monitoring survey from October 2017 to April 2018. It was a community-based study in which the sampling was done using the 2011 census. A probability-based sampling system was adopted in the form of a stratified multistage geographical cluster. The study covered the age group between 15 to 69 [30]. The availability of essential medicines and technology that the WHO-PEN( Package of Essential Noncommunicable Disease Interventions) recommended was also assessed, and secondary facilities were followed as per the definitions of NPCDCS [31]. NPCDCS was implemented in most of the CHCs (Community Health Centers) (72.8%). There was no significant difference in the indicators in the districts and towns where NPCDCS was implemented versus in those where it wasn't. In the case of Diabetes, the essential medicines were only available in one-fifth of the total Primary Health Centers, about three-fifths in the private hospitals, and nearly half in the Community health centers. Insulin availability was about half in CHCs and only 21.4% in PHCs. These gaps in the provision of health services and medicines and the absence of availability of proper equipment have already been pointed out by other researchers as well [32].

Yet another study was conducted on the implementation of NPCDCS in the district of Udupi, Karnataka. Udupi was one of the first districts to be included by the NPCDCS in its second phase (the year 2010-12). The NDC clinics were implemented in the CHCs by 2016, and most of the staff was recruited by that time [33]. The study observed some obstacles in the implementation of NPCDCS. For instance, in the sub-centers, the healthcare workers have been burdened with several other national health programs along with NCD programs like NPCDCS, which makes it difficult to focus on NPCDCS-related activities. The majority of the respondents raised the issue of lack of budget. This puts a hold on the future planning and execution of the program. It makes it hard to procure essential medicines and adequate technology in time with a low budget and delays transactions [34].

Discussion

The findings of the various studies carried out over the period of after 2010 found that the decrease in case burden of Diabetes has not been as significant as it was aimed to be. Out of the various studies mentioned above from different parts of the country, in the city of Jaipur, it was found that only 6,674, which is 48% of the total population, have been screened for NCDs. In the state of Gujrat in 2013, the total coverage of screening the target population was much lower, i.e., 25%. In a more recent study conducted from May 2019 to September 2020, the number of screened people increased but after that decreased in the following months. Similar findings were presented in the Gandhinagar district in Gujarat, which underwent a similar study [35].

Similarly, a research effort was undertaken on the southern coast of India, revealing a notably lower coverage percentage. Among the 36 subcenters examined, 13 exhibited a screening rate of 20-30%, while two subcenters had a screening rate below 10%. It is also noticed that the program has a very less screening rate and coverage area, the changes in the diabetes cases are difficult to assess when majority of the target population is undetected [36].

In the Belagavi taluka district context, the study encompassed 36 health centers. Within the population, comprising 65,096 individuals aged 30 or above, screenings for both Diabetes and hypertension were conducted. The study identified 3124 individuals who tested positive for Diabetes, yielding a prevalence rate of 4.87%. This rate was below the global diabetes prevalence of 8.5% among individuals aged 18 years or older, as reported by the WHO in 2014 [37]. In contrast, a separate study in rural areas of Karnataka reported a higher prevalence rate of 22%. The case burden was not decreased as significantly as it was initially intended [38]. A concurrent study spanning 15 states and published in 2017 indicated rural area diabetes prevalence rates of 7.3% and 5.2% [39].

In the Udupi district study, a significant obstacle to the NPCDCS implementation was the need for more healthcare personnel and hospital staff this was also reflected in their management of cases of Diabetes. There is a pressing need for finance in healthcare in India, as 80% of the healthcare is provided by the private sector. With the efforts of the Karnataka government, the healthcare preference towards private health institutes is recorded below 60%. The vulnerable population should receive insurance, and primary care for NCD treatment should be prioritized [40]. One of the primary barriers to NPCDCS implementation stems from numerous vacant positions within Community Health Centers and sub-centers. Despite regular Information, Education, and Communication (IEC) activities, awareness of the program needs to be improved, particularly in rural areas [41].

Himachal Pradesh conducted screenings in three districts, encompassing 249,696 individuals, of which 5.72% tested positive for Diabetes. Evidently, the limited NPCDCS implementation is linked to the growing disease burden within the population [42]. Critical deficiencies include inadequate screening coverage and insufficient technical proficiency among healthcare workers [43]. Human resources play a pivotal role in successfully operating national health programs. National NCD monitoring survey data reveals an improper posting policy for doctors, misaligned with their training, which was previously documented. Insulin availability was about half in CHCs and only 21.4% in PHCs. These gaps in the provision of health services and medicines and the absence of availability of proper equipment have already been pointed out by other researchers as well [44].

While India continues to address the primary NCDs - Diabetes Mellitus, Cardiovascular Diseases, and Chronic Renal Disease - urgent attention is also required for other NCDs, including cancer, mental health issues, and chronic renal diseases. In a strategic shift, India has incorporated NCDs into its comprehensive primary healthcare approach for universal health coverage. Primary Health Centers and sub-centers are being transformed into Health and Wellness Centers (HWCs), emphasizing personnel augmentation, essential medical supplies, and technological advancements [45]. Recruitment and retention strategies have been adopted to address the challenges mentioned above, including utilizing virtual learning through Massive Open Online Courses (MOOCs) [46]. Since 2018, spirometers for diagnosis, pulse oximeters, and nebulizers for management and monitoring have been introduced under the NPCDCS and WHO-PEN guidelines [47]. Table 1 depicts various NCDS surveys and cross-sectional studies in India.

**Table 1:** Findings and conclusions of various NCD surveys and cross-sectional studies NCD: Non-Communicable Diseases; NPCDCS: National Program for Prevention and Control of Cancer, Diabetes, Cardiovascular Disease, and Stroke; OPD: Out Patient Department; ANM: Auxiliary Nurse Midwife; SES: Socio-Economic Sectors

Author	Method of study	Area (District)	Findings	Conclusion
Mohan V, Shanthirani S, Deepa R et al. [22]	Epidemiological study (2001)	Chennai	The socioeconomic position and way of life of the people living in the two locations differed significantly.	There was a notable rise in the relative odds ratio for both diabetes and impaired glucose tolerance.
Sadhana Meena et al. [27]	Observational study (2019)	Jaipur	48% (6674/13917) of individuals older than 30 years were screened. 51.5% of those who were screened had one or more NCDs (13.5% were freshly screened for NCD)	The study concludes that the implementation of NPCDCS program activities is not in synch with the health staff awareness level.
Kashyap VH, Shivaswamy MS [29]	Cross-sectional study (2018)	Belagavi taluka	The prevalence of diabetes was found to be 4.87%, 64,096 people were screened, which equals a coverage rate of 25.77% across all 36 subcenters. Diabetes is determined to be 4.87% prevalent.	The subcenters conducted NCD screenings, however only 69% of the ANMs possessed the required training and tools.
Prasant Mathur et al. [30]	National level cross sectional survey (2017)	All 29 states of India	The poll was completed by 10659 adults and 11139 homes in total. 9.3% of people had elevated blood glucose.	The awareness and equipment grossly lack in the rural areas.
Bhavesh Modi et al. [35]	Secondary Data from CHC and NDC cell (2015)	Gandhinagar, Gujrat	60.7% of the district's positions were unfilled overall. The population of 90399 had a diabetes prevalence of 9.9%.	While human resources are sufficiently accessible at the district level, they are egregiously deficient at the sub-district level, which accounts for the subpar outreach and OPD actions there.
Ranjit Mohan Anjana et al. [39]	Cross sectional study (2016)	15 states of India, Bihar, Punjab, Tamil Nadu, Chhattisgarh etc.	Diabetes was present in 73% of India's 15 states as a whole. Diabetes was more common in urban regions (11%) than in rural areas (5%) and in mainland states (8%) than in the northeast (59%), ranging from 4% in Bihar to 10% in Punjab.	A higher frequency of diabetes was found among low SES groups in the metropolitan areas of the more economically developed states, our findings indicate an epidemiological transition.
Bhattacharyya D, Pattanshetty SM, Duttagupta C [40]	Cross sectional study (2015)	Udipi, Karnataka	A 98.02% response rate from 397 households out of the 405 total households took part in the study. In morbidity responders, diabetes accounts for 21.7%.	Both the actual and perceived demands for health care were comparable in both rural and urban locations, and there was no apparent difference in the nature of needs for diabetes among both.

## Conclusions

In conclusion, it is fair to say that the NPCDCS has achieved a considerable level of implementation in recent years but severely lacks awareness and working staff, especially in the rural areas and the southern part of the country. It has been made clear that the IEC activities are deficient, and more work is to be done to promote behavioral changes and the adoption of healthier lifestyle options. The reporting of NDCs does not match the factual data collected in the cross-sectional surveys. The National NCDs Monitoring Survey has reported significant gaps in the NPCDCS response to NCDs. Some of them are the unavailability of essential medicines, technologies, and adequate training of the health care workers. This survey can serve as a benchmark or threshold for future assessment of the coverage of NCDs. The study in Belagavi has shown a reduced prevalence of Diabetes, even less than the global prevalence rates. However, the study also reports a grave requirement for follow-up treatment services of the screened-positive individuals in the subcentres under NPCDCS.
